# Epigenetic Alterations Induced by Smoking and Their Intersection with Artificial Intelligence: A Narrative Review

**DOI:** 10.3390/ijerph22111622

**Published:** 2025-10-24

**Authors:** Edith Simona Ianosi, Daria Maria Tomoroga, Anca Meda Văsieșiu, Bianca Liana Grigorescu, Mara Vultur, Maria Beatrice Ianosi

**Affiliations:** 1 Department of Pulmonology, University of Medicine, Pharmacy, Science and Technology “George Emil Palade” of Târgu Mureș, 540139 Târgu Mureș, Romania; edith.ianosi@umfst.ro (E.S.I.); mara.vultur@umfst.ro (M.V.); 2 University of Medicine, Pharmacy, Science and Technology “George Emil Palade” of Târgu Mureș, 540139 Târgu Mureș, Romania; 3 Department of Infectious Disease, University of Medicine, Pharmacy, Science and Technology “George Emil Palade” of Târgu Mureș, 540139 Târgu Mureș, Romania; anca-meda.vasiesiu@umfst.ro; 4 Department of Anaesthesiology and Intensive Care, University of Medicine, Pharmacy, Science and Technology “George Emil Palade” of Târgu Mureș, 540139 Târgu Mureș, Romania; bianca.grigorescu@umfst.ro; 5 Clinic of Pulmonology, County Hospital Mures, 540011 Târgu Mures, Romania; ianosi.maria-beatrice@stud18.umfst.ro

**Keywords:** epigenetic inheritance, DNA methylation, histone modification, non-coding RNA, transgenerational respiratory disease, multigenerational respiratory disease, artificial intelligence

## Abstract

Introduction: Cigarette smoking is unquestionably associated with an increase in morbidity and mortality worldwide, exerting significant adverse effects on respiratory health. The impact of tobacco persists in the epigenome long after smoking cessation. Furthermore, the offspring of smokers may also be affected by the detrimental effects of smoking. Material and methods: The modifications made to the body, such as DNA methylation, histone modification, and regulation by non-coding RNAs, do not change the DNA sequence but can influence gene expression. In respiratory disease, multigenerational effects have been reported in humans, with an increased risk of asthma or COPD and decreased lung function in offspring, despite them not being exposed to smoke. Prenatal nicotine exposure leads to pulmonary pathology that persists across three consecutive generations, supported by animal studies conducted by Rehan et al. Significant advances in high-throughput genomic and epigenomic technologies have enabled the discovery of molecular phenotypes. These either reflect or are influenced by them. Due to the hidden environmental effects and the rise of artificial intelligence (AI) in biomedical research, new predictive models are emerging that not only explain complex data but also enable earlier detection and prevention of smoking-related diseases. In this narrative review, we synthesise the latest research on how smoking affects gene regulation and chromatin structure, emphasising how tobacco can increase vulnerability to multiple diseases. Discussion: For many years, it was widely believed that diseases are solely inherited through genetics. However, recent research in epigenetics has led to a significant realisation: environmental factors play a crucial role in an individual’s life. External influences leave a mark on DNA that can influence future health and offer insights into potential illnesses. In this context, it is possible that in the future, doctors might treat people not as a whole but as individual beings, with personalised medication, tests, and other approaches. Conclusions: The accumulated evidence suggests that exposure to various environmental factors is associated with multigenerational changes in gene expression patterns, which may contribute to increased disease risk. The application of artificial intelligence in this domain is currently a crucial tool for researching potential future health issues in individuals, and it holds a powerful prospect that could transform current medical and scientific practice.

## 1. Introduction

Indirect or passive smoke exposure is linked to about 1.3 million of the roughly 8 million tobacco-related deaths globally each year [1].

Alongside a growing recognition of its generational consequences, tobacco smoking exerts harmful effects on the respiratory, cardiovascular, and vascular systems, among others. The concept of disease pathophysiology has advanced significantly by exposing the toxicological relationships that reveal the cause-and-effect relationship between diseases caused by tobacco use and the organism, with the biological basis lying in epigenetic inheritance (the inheritance of phenotypic traits through molecular mechanisms without involving DNA sequence) [2].

In fact, population-based studies have documented epigenetic changes in response to various environmental factors, including nutrition, psychological stress, pollutants, and tobacco smoke. All of these approaches can induce epigenetic modifications to DNA itself (DNA methylation) or its associated histone proteins (chromatin remodelling) [3]. Additionally, a small number of epigenetic marks can evade the widespread reset during gametogenesis and early embryonic development, allowing them to be inherited across generations [4].

Asthma and COPD have traditionally been viewed as the result of a combination of genetic susceptibilities and an array of environmental exposures. Longitudinal animal models and human studies confirm that the phenotypes relevant to respiratory outcomes in progeny can be influenced by epigenetic mechanisms established through exposure of ancestors to environmental influences [5,6]. This affirmation highlights the fact that a future improvement of public health strategies should include the dissemination of knowledge regarding the molecular determinants of environmental risk.

## 2. Material and Methods

This is a narrative review. We designed this review as an integrative synthesis, aiming not only to compile the literature but also to critically examine the epistemic ideas underlying contemporary research on the epigenetics of smoking and its connection with machine learning models [7].

Relevant studies were identified through searches in PubMed (U.S. National Library of Medicine, Bethesda, MD, USA), Google Scholar (Google LLC, Mountain View, CA, USA), and Web of Science (Clarivate, Ann Arbor, MI, USA), focusing on peer-reviewed articles, systematic reviews, and original research on the epigenetics of smoking, its effects on the organism, and the intersection with artificial intelligence. Clinical terms (e.g., COPD, asthma), molecular descriptors (DNA methylation, histone acetylation), and computational vocabulary (machine learning, artificial intelligence) were used in different combinations to cover overlapping domains and highlight the interdisciplinary nature of this field [8].

### 2.1. Selection of Relevant Studies

Studies were selected for their relevance to mechanisms and illustrative AI applications. Priority was given to longitudinal and multigenerational designs, which enable inference of inheritance patterns [9]. Additionally, we identified whole-genome or high-throughput findings that reflect changes across the entire epigenome rather than at single loci [10], and materials that demonstrate the capacity of AI technologies to scan and search the epigenome for variations associated with disease risk [11,12,13]. Studies were prioritised if they provided illustrative findings on multigenerational inheritance, high-throughput approaches, or AI applications.

### 2.2. Review Approach

The approach taken in this review focuses on the scientific understanding that epigenetic inheritance and AI are increasingly interconnected areas of study with the potential to transform the world as we know it. This focused perspective was undertaken to produce a descriptive review, as well as to develop a critical repertoire that highlights the importance of the current knowledge in this field, which must be integrated into everyday medicine for the prediction and prevention of diseases [14].

Literature was organised for discussion across the main sub-fields outlined in the paper: DNA methylation, histone modification, non-coding RNAs, experimental animal studies, paternal epigenetic transmission, and the application of artificial intelligence to identify epigenetic signatures.

### 2.3. Smoking-Associated Respiratory Diseases Caused by Epigenetic Mechanisms

Tobacco exerts a direct and significant pathobiological impact, rather than merely being a general health risk. Cigarette smoke is composed of more than 7000 chemical compounds, including heavy metals, nitrosamines, and polycyclic aromatic hydrocarbons (PAHs), acting as potent epigenetic modulators. This allows these agents to directly affect the activity of various enzymatic systems that regulate DNA methylation, histone modification, and chromatin structure [15,16].

### 2.4. DNA Methylation

DNA methylation involves the covalent addition of methyl groups to cytosine bases, mainly occurring on CpG islands. It is one of the most vital strategies for altering gene expression without modifying the DNA sequence [17]. Methylation generally takes place in promoter regions, where it silences the gene associated with that region. A subset of gene promoters in the airway epithelium of smokers shows both increased and decreased methylation. While hypermethylation can lead to gene silencing and tumour suppression, hypomethylation may cause genomic instability and cancer-specific changes [18]. Some of these alterations can increase the patient’s risk of lung cancer or breathing issues.

Notably, germline cells (sperm and oocytes) have the same methylation pattern. In other words, certain epigenetic marks can persist in germ cells and have been linked with inherited phenotypes [19]. Studies have also suggested that if a mother smokes while pregnant, the offspring may suffer from epigenetic changes, depending on where the affected genes are located (which could relate to abnormal methylation). This reveals that smoking could not only be bad for the smoker but also for their progeny and generations to come.

Significantly, inflammation-related, airway remodelling, and pulmonary function-associated genes were found to be differentially methylated. Examples include ADAM33 [20], IL13 [21], and SERPINA1 [22]. Table 1 summarises the key genes where smoking has left identifiable epigenetic marks, linking them to disease risks that recur in both clinical and experimental settings. The long-lasting memory of ancestral exposure is carried both by the exposed individuals themselves and in their descendants, the latter of whom were never exposed to the toxicants [5,23].

In a large Lifelines cohort, grandchildren of women who smoked during pregnancy exhibited altered DNA methylation at asthma-related loci, even if their parents did not smoke. The results suggest that methylation changes caused by smoking can have long-lasting effects on disease risk through mechanisms that are yet to be identified [24].

It is increasingly likely that the seemingly transient gene expression changes in response to smoking-induced methylation are actually a crucial epigenetic pattern and can predispose later generations to respiratory illness. This information is vital from a public health perspective because it illustrates how tobacco use results in multigenerational effects. This is something to keep in consideration when establishing policies that are meant to lower smoking rates [25].

Table 1 Key genes and epigenetic modifications induced by cigarette smoking, along with their associated disease risks. Some of these genes will be discussed in more detail in the following sections to highlight their roles in selected diseases and possible multigenerational effects.

### 2.5. Histone Modifications

These proteins bind to DNA and assemble into larger structures called nucleosomes, the fundamental physical units of chromatin. These histones can be acetylated, methylated, or phosphorylated, and this changes how densely packed the DNA is. A loose chromatin structure facilitates access to genes by the transcription machinery, resulting in enhanced gene expression.

It has been shown that tobacco smoke is linked to the acetylation of histone H3 at lysine 9 in airway epithelial cells, thereby promoting the activation of pro-inflammatory genes and leading to a chronic inflammatory state in the lung [26]. It is essential to highlight recent research findings: changes in histones are not merely somatic-specific, and the modifications that germline cells undergo can survive the extensive epigenetic reprogramming after fertilisation. These changes can influence gene expression during lung development in the offspring and even in subsequent generations, if they persist [27]. This idea offers yet another way in which smoking-induced damage could be biologically “regarded” and transmitted, affecting respiratory health long after the initial exposure.

### 2.6. Non-Coding RNA Regulation

Non-coding RNAs, representing a significant number of transcripts found in mammalian cells, include long non-coding RNAs (lncRNAs) and microRNAs (miRNAs), which are key elements in regulating gene activity at the post-transcriptional level following the generation of prototypical RNA from a gene [28]. They are also capable of serving as molecular fine-tuners to ensure that proteins are produced at the appropriate time and in the correct quantity.

Cigarette smoke can disrupt this balance by influencing miRNA expression related to inflammation and alterations in lung tissue structure [29]. The presence of smoke near the lungs may elevate levels of miR-223 and miR-21, two signalling molecules that regulate pathways determining airway responsiveness (to inhaled irritants, allergens, or inflammatory signals) and the extent of scar tissue formation. If such expression patterns occur in germline precursor cells (as shown in animal models), they could contribute to effects observed in subsequent generations [30].

### 2.7. The Rehan et al. Study

Besides the common knowledge that maternal smoking during pregnancy has a significant effect on the development of the fetus, leading to cardiac problems and low birthweight [31,32], experimental evidence in rodents provides strong support for the hypothesis that in utero exposure to smoking can produce transgenerational alterations of lung structure and function. Rehan et al. described a groundbreaking rat study, which demonstrated impaired lung function and the adverse effects that smoke exposure has had across generations [33]. For the F1 offspring, gestational smoking exposure led to decreased alveolar surface area and increased pulmonary structural abnormalities, such as thickened alveolar septa with prominent collagen deposition and impaired elastin fibre assembly [33]. Additionally, suppression of peroxisome proliferator-activated receptor gamma (PPAR-γ) signalling, a pathway essential for normal alveolar development, occurs alongside these changes. Remarkably, when the F1 offspring (which had never been exposed to cigarette smoke after birth) were bred, their next generation (F2) also developed similar pulmonary pathology. However, there was no direct exposure to smoke during their own gestation [33]. This suggests that the phenotypes observed were transmitted via the germline, most likely through heritable, stable epigenetic modifications rather than DNA sequence changes.

The interpretation that epigenetic responses are transmitted from sperm to offspring and through germline passage in a transgenerational manner is emphasised in Leslie’s review of multigenerational nicotine effects. The article highlights the persistence of epigenetics across generations, suggesting that smoke-induced epigenetic changes can evade both significant reprogramming waves—first during gametogenesis and then in early embryo [34]. These stable marks—which may include DNA methylation, histone modifications, and changes in non-coding RNA profiles—serve as a ‘molecular memory’ or legacy of environmental exposure that influences the development of lung remodelling episodes many years after the initial exposure.

**Figure 1:**
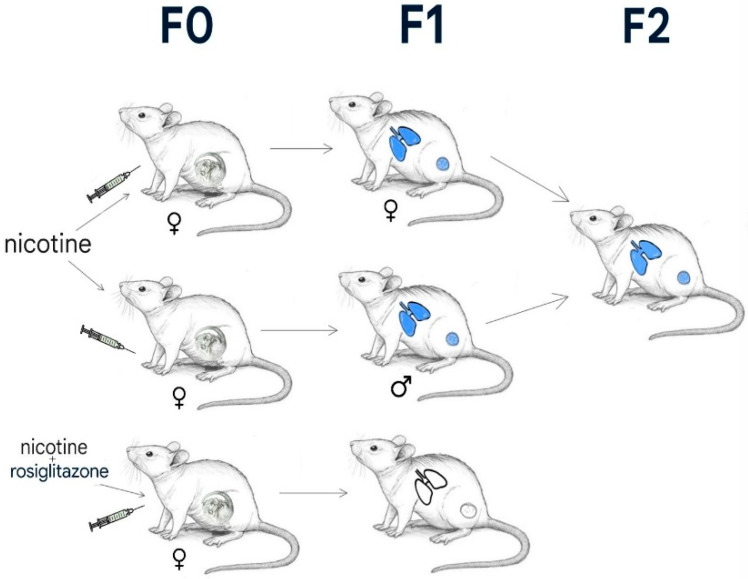
The illustration shows the experimental approach and results of Rehan et al. The F0 generation received nicotine (the first two groups) or a combination of nicotine and rosiglitazone (the last one). The lungs and gonads of male and female offspring (F1 generation) display epigenetic changes, with the lungs showing an asthma-like functional phenotype (blue nicotine-induced modifications). The offspring of mice treated with nicotine and rosiglitazone do not show these nicotine effects. The F2 generation, the offspring of F1 mated pairs, demonstrates similar smoke-induced changes in lung function as their ancestors, despite not being exposed to the substance [34].

Notably, Rehan et al. have demonstrated that pharmacological activation of PPAR-γ during gestational nicotine exposure prevents both the structural lung defects in directly exposed offspring and their inheritance in the F2 generation (Figure 1).

Each of these results provides the first evidence in a mouse model that specific molecular abnormalities contribute to the gene-environment interactions underlying the transgenerational inheritance of pulmonary morbidity, collectively highlighting a key principle of this study. Tracking complex environmental events through equally detailed molecular marks in germ cells can significantly influence susceptibility to diseases among offspring who have never themselves been exposed to a particular harmful agent [33,34].

### 2.8. Paternal Smoking and Epigenetic Transmission

Emerging evidence has further illustrated how paternal influences, notably smoking, can epigenetically program the developing placenta and thereby affect fetal health in later life. Bhadsavle and Golding (2022) [35] have published compelling rodent data demonstrating that paternal stressors can epigenetically modify sperm, especially impacting imprinted genes vital for placental development and fetal growth. These sperm-borne epigenetic marks were shown to disrupt placental architecture, leading to significant alterations in trophoblastic zones and histological organisation; thus, making the offspring susceptible to metabolic, developmental, and respiratory dysfunctions.

Similarly, Vlachou et al. (2025) demonstrated that smoking by parents, or even grandparents, results in inheritable changes mainly through alterations in DNA methylation affecting germ cells and placental tissues, thereby modulating susceptibility not only to early-onset respiratory problems but also to metabolic diseases [36].

### 2.9. AI in Epigenetics

Artificial intelligence (AI) is increasingly applied to high-dimensional epigenomic data and shows promise for pattern discovery. In combination with epigenetics, it has recently become one of the most powerful tools in modern science.

Because epigenetic marks can be reversible and highly dependent on context, they serve as strong biomarkers for disease susceptibility [37]. Genomic and epigenomic datasets are inherently high-dimensional, typically comprising millions of features per individual. The nonlinear interactions and complex patterns within this data are likely to make traditional statistical methods ineffective. The ability to classify epigenetic states—not only across different cell types and tissues throughout the body but also in predicting an individual’s future disease risk—is where AI can be most beneficial [38,39].

Both fields are merging and transforming medicine as well. Combining AI-driven analytics with epigenetic data is now close to predicting who could develop anything from cancer and heart problems to lung disease, sometimes decades before their symptoms appear.

### 2.10. The Molecular Memory of the Genome

Variables such as the environment and genetic factors may influence epigenetic imprinting mechanisms. Of particular importance is the observation that some of these marks persist during gametogenesis or early embryogenesis. Such modifications pass a person’s legacy across generations.

This effect on disease inheritance is evident in models showing that paternal smoking alters sperm methylation patterns. The placental development of the next generation may be negatively affected by in utero damage, leading to a higher risk of respiratory problems later in life for those offspring [25,36,40]

AI algorithms have been applied to DNA methylation landscapes in research studies, showing potential to identify early indicators of respiratory diseases (e.g., asthma or COPD) when no symptoms are yet detectable [37,41]. Additionally, machine learning models can predict histone modification states and provide insights into gene expression patterns that influence lung function [13,34]. Moreover, advanced deep learning methods can identify abnormal miRNA and lncRNA signatures, which act as predictive biomarkers for pulmonary inflammation and tissue remodelling [42].

### 2.11. Risk of Bias

Across the included studies, most adjusted for key covariates such as age, sex, and cell-type composition. Several studies also accounted for smoking intensity and cessation status, and implemented batch effect correction. However, socioeconomic status (SES) and environmental co-exposures were often incompletely measured or not included in the models. Few studies performed ancestry-stratified analyses.

As a result, residual confounding is likely and may influence both estimated effect sizes and model generalizability. Transparent reporting and harmonisation of covariate adjustment strategies across studies are essential for improving reproducibility.

## 3. Disease Prediction

### 3.1. Respiratory Disease Prediction

The concept of epigenetic memory provides a robust framework for understanding how tobacco exposure leaves lasting molecular epigenetic markers that are transmitted across generations, with consequences for the respiratory health of subsequent generations [33]. Recent research suggests that changes to the epigenome in the germ line (such as defects in DNA methylation or histone modification) can avoid reprogramming during fertilisation and contribute to the persistence of disease-related signatures. These findings complement a growing body of research indicating that tobacco-smoking-induced alterations to the epigenome of sperm result in changed function of the placenta and the viability of trophoblast cells, which directly affect respiratory vulnerability in utero [43].

At the same time, artificial intelligence offers unique synergies that enable the integration of high-dimensional epigenetic datasets, such as methylation landscapes, and is capable of identifying predictive biomarkers for lung dysfunction [37]. Today’s learning methods can identify individuals at risk for multiple respiratory diseases, and even early-age lung cancer, with high accuracy in specific cohorts, paving the way for preventative treatments that might reduce the multigenerational burden of tobacco [42,44].

### 3.2. Neurological Disease Prediction

Epigenetic modifications are increasingly recognised for their role in regulating gene expression in the brain. Abnormalities in these mechanisms have been associated with various neurological and psychiatric disorders, such as Alzheimer’s disease, Parkinson’s disease, schizophrenia, Rett syndrome, and others [45,46].

Nicotine, one of the most common psychoactive drugs, exerts a powerful influence on the brain’s reward system, and increasing evidence suggests an epigenetic basis for the health risks. Chronic nicotine use has been shown to cause histone modifications, especially acetylation, which then influence gene expression patterns in reward regions like the nucleus accumbens. These epigenetic changes result in the upregulation of genes such as FosB, a key transcription factor involved in neuronal plasticity, leading to heightened reactivity of dopaminergic circuits to future stimuli. Kandel & Kandel highlight in their study how epigenetic modifications within the NAcc (nucleus accumbens) may contribute to dysregulated dopamine signalling, supporting increased reward-responsiveness and providing a neurobiological foundation for nicotine as a gateway drug [47].

Additionally, adolescence is a crucial period characterised by increased vulnerability. Dao et al. examined nicotine exposure during this developmental stage and identified changes in the dopamine system, along with lasting effects on reward-related behaviour [48].

In light of these findings, we can affirm that early life nicotine exposure reprograms the nervous system through epigenetic mechanisms, while enhancing the brain’s reward system and posing risks to dopaminergic pathways.

### 3.3. From Prediction to Prevention

AI offers a practical promise in epigenetics: prediction can lead to prevention. AI is used in oncology, for instance, to classify tumours based on their methylation profiles, which can aid in identifying the most effective treatment.

Building on the idea that AI can turn epigenetic prediction into prevention, recent advancements have demonstrated its significance in early-life prediction and prevention. AI models utilising epigenetic data can identify individuals with heritable epigenetic damage caused by maternal smoking, enabling early preventative measures to lower the risk of respiratory issues in children of these mothers.

A study by Rauschert et al. developed a DNA methylation score that effectively indicates fetal exposure to maternal smoking during pregnancy. This risk score surpassed previous models, demonstrating the potential of epigenetic biomarkers for predicting the health effects of maternal smoking [49].

Furthermore, Zakarya et al. examined the impacts of in utero exposure to maternal cigarette smoke (MCS), environmental tobacco smoke (ETS), and electronic cigarette vapour (ECV) on fetal and postnatal lung function, as well as epigenetic modifications. Their findings emphasise the importance of considering all forms of tobacco exposure when assessing respiratory health risks in offspring [50].

Together, these studies underscore the potential of integrating artificial intelligence with epigenetic biomarkers to forecast infants at risk due to maternal smoking.

### 3.4. Challenges and Ethical Considerations

Introducing AI into epigenetic risk prediction has significant scalable clinical potential, but it also raises equally critical ethical questions. One aspect of this is especially notable, as AI systems may perpetuate disparities if specific ancestries are excluded from the training data [51].

Beyond scholarly novelty, applying AI to epigenetic risk prediction has practical implications. For instance, in the absence of stringent regulation, prenatal screening based on epigenetic profiles may inadvertently stigmatise parents or be abused by insurance companies. Consent should specifically address the potential for future reuse or reanalysis of methylation data, and models should report uncertainty intervals rather than absolute risk scores to avoid such outcomes.

Clinically, the ultra-rapid sequencing studies—such as Ashley’s work at Stanford that demonstrated genome analysis could be completed within 24 h—not only highlight its transformational potential but also raise ethical concerns: from rushed consent to opaque decision-making by automated systems [52]. Ashley emphasises the need for transparency, clarity about scope, and family-inclusive counselling, especially because epigenetic risk often impacts future generations [53]. The necessity of non-blaming communication is equally crucial; in order to prevent moral or social repercussions for individuals, results should be presented as “associated risks” as opposed to deterministic outcomes. Additionally, participants must be able to withdraw, particularly since information about epigenetic risk can be passed down through generations. Ensuring fairness is an essential ethical safeguard. AI systems can maintain inequities if underrepresented ancestries are omitted from training datasets; consequently, performance must be verified across various ancestry groups and disclosed transparently.

Consequently, integrating AI with epigenetic medicine demands a framework founded on dynamic consent, privacy-protecting governance, and fairness assessments [51,54,55,56,57,58,59].

### 3.5. Future Perspectives

The integration of epigenetics and AI into everyday life has the potential to revolutionise the way we predict and prevent diseases. New techniques, such as EPIC-Seq, may enable non-invasive, high-resolution profiling of gene expression through DNA sequencing, offering an opportunity for early detection of diseases affecting the respiratory system, and not only [60].

Moreover, progress in longitudinal AI models, such as multi-mean Gaussian processes, enables the prediction of DNA methylation changes over time, supporting the prediction of disease course and the development of personalised interventions [7].

Furthermore, the application of AI to epigenetic data is also shaping precision medicine, particularly in the field of cancer care. According to the AI algorithms currently employed to guide the use of genetic risk factors, the goal should be to progress toward disease screening, thereby enabling the forecasting of patient outcomes [8,9].

These technological advances showcase the potential of AI-powered epigenetic research to revolutionise our understanding and treatment of respiratory diseases, especially those impacted by environmental factors.

## 4. Discussion

The objective of this review was to summarise how smoking induces epigenetic changes and how AI can be used to detect and predict smoking-related disease. Alongside a substantial body of evidence from human and animal studies, the conclusions in this current study represent a significant advance in our understanding of how environmental exposure (e.g., cigarette smoke) affects the respiratory tract. For decades, the prevailing view was that diseases like asthma and chronic obstructive pulmonary disease could be almost entirely explained by two factors: either a person inherited a genetic predisposition or they were directly exposed to harmful environmental substances. However, recent discoveries in the emerging field of multigenerational epigenetic inheritance are challenging these simplistic perspectives.

Exposure is central because harmful substances rarely remain localized and can affect the entire organism. Instead, they spread and cause effects throughout the entire organism. They can leave behind biological marks at the level of the genome—marks that do not alter the DNA itself but that influence how genes are turned on or off. These marks, called epigenetic modifications, can last long after the original exposure has disappeared and, troublingly, can be passed between generations. There are many examples of these prints above the genome, but one of the most well-known is DNA methylation.

### 4.1. The Importance of DNA Methylation

Lately, new high-throughput studies have reinforced the association between methylation and pulmonary phenotypes. A key finding has emerged around the AHRR gene: the methylation site cg05575921 in this particular gene has been identified as the most reliable molecular signature of tobacco smoke exposure, enabling the addition of precision to risk models for lung cancer screening [61,62,63]. Building on this information, recent epigenome-wide studies confirm that respiratory AHRR methylation is not merely a fixed marker of exposure. It also has the power of showing a clear dose–response pattern with the amount smoked, has intermediate levels in ex-smokers, and, surprisingly, can partially reverse over time. More interesting is how lifestyle factors, such as physical activity and sleep, influence the rate of epigenetic recovery, emphasising their potential as crucial biomarkers. Moreover, these indicators can monitor changes in respiratory risk after the smoker has quit tobacco [64].

In humans, a direct analysis of the epigenome in lung tissue suggests that smoking produces a distinct methylation signature in the lung. Stueve et al. identified a strong correlation between lung and blood methylation signatures and further documented lung-specific smoke-inducible enhancers. Among the top-ranking loci [65,66], AHRR has proven to be a significant, universally smoke-responsive site in the lung tissue, indicating local regulatory activity relevant for pulmonary inflammation [67].

### 4.2. The HUNT Study

Within the framework of the HUNT Study, the researchers examined the effects of tobacco on DNA methylation and the potential implications for pulmonary health. They performed repeated measurements of DNA methylation over twenty years. The findings indicated that smoking is associated with lasting changes in the blood epigenome and that these modifications remain stable over time. This emphasises the enduring impact of tobacco on the epigenetic landscape. The researchers employed Mendelian randomisation to assess causal relationships, finding that smoking affects changes in DNA methylation. These findings highlight the complexity of genetic regulation and demonstrate how epigenetic profiling can be utilised to monitor environmental exposures. This research opens new avenues for investigating the intricate relationship between lifestyle factors, epigenetic modifications, and disease development, laying the groundwork for future preventive and predictive strategies in medicine [68].

### 4.3. The Future with the GrimAge Clock

Accumulating evidence indicates that the use of DNA methylation-based biomarkers, particularly a new technology called GrimAge, which is an epigenetic clock, has significantly enhanced our understanding of biological ageing. GrimAge is a composite biomarker consisting of DNA methylation-based surrogates for plasma proteins, cumulative smoking exposure, and predictions of lifespan and healthspan. It indicates superior predictive ability for diseases and cancer compared to other epigenetic clocks. This improvement results from including DNA methylation predictors of physiological risk factors and stressors, such as adrenomedullin, C-reactive protein, plasminogen activator inhibitor-1, and growth differentiation factor 15, into the algorithm [69]. A newer version, GrimAge version 2, incorporates additional markers and is more effective at predicting multiple health-related outcomes, including type 2 diabetes and visceral fat, making it a more accurate measure of biological age [70].

The integration of artificial intelligence into epigenetic clocks broadens their range of applications. Complex methylation data, along with lifestyle and health information, can be analysed by AI models to generate personalised predictions of biological age and to assess health risks. For example, AI-based models, such as ExplaiNAble BioLogical-ENABL-Age, have been developed to forecast epigenetic age acceleration by considering modifiable factors, including patient histories and environmental exposures. These advances enable a more detailed understanding of the ageing process and support the development of interventions aimed at preventing age-related diseases [71,72]. It has also been reported that smoking influences epigenetic age acceleration in lung bronchoalveolar lavage (BAL) cells. In this context, GrimAge indicates smoking-related acceleration of biological age in lung cells. Furthermore, the specific effect was observed in patients with multiple sclerosis: scientists demonstrated transcriptional differences between the cells of smokers and non-smokers. This highlights the tissue-specific effects of smoking on epigenetic ageing and emphasises the importance of considering tissue-specific biomarkers in ageing research [73,74].

Regarding this aspect, the emergence of transgenerational epigenetic inheritance challenges the conventional notion that diseases such as asthma and COPD are entirely genetically determined or directly caused by environmental factors. For instance, the work conducted by Rehan et al. clearly indicates the transgenerational impact of in utero nicotine exposure in generation F0, leading to increased disease susceptibility across subsequent generations. Because of this epigenetic legacy, the effect of smoking is now known to extend far beyond the smoker, putting the lives of unborn family members as well as individuals in proximity at risk.

While mutations become permanent changes to the DNA sequence, epigenetic changes can be reversed but remain stable enough to be passed on through cell divisions and even down the germline to the next generation. The effects of such persistence are significant. It is also a response to the fact that what used to be regarded as a personal risk factor, smoking, is now widely and correctly seen as something that is imposed on others, apart from the individual smoker. The harm takes hold in the family tree, affecting the health of future generations as well. Therefore, the impacts are not just personal but societal—entire populations could face a greater burden of disease as a result of their ancestors being exposed to noxious substances.

In addition to addressing the risk of current adverse health effects, public health efforts should highlight the long-term consequences of tobacco use. The finding that epigenetic changes are somewhat reversible is an excellent target for intervention, since it means that smokers could potentially avoid the health downsides of smoking through drugs or lifestyle changes. This paves the way for interventions that might mitigate the deleterious effects—not just for smokers themselves, but for their children and grandchildren.

### 4.4. Past, Present and Future Potential

The knowledge we have today about genes and their influence on our lives was made possible through the Human Genome Project (HGP), which started in 1990 and finished in 2003. Its main goal was to tackle the challenges of modern biology by creating a complete reference sequence of human DNA. This transformed the landscape of biological research, affecting how it is funded, organised, and carried out. A draft of the genome was published by 2000, and a nearly complete sequence was available within the following three years [75]. By providing a reference genome, the HGP laid a foundation for many future genomic initiatives [76]. The project also raised important ethical and philosophical questions. From the start, it had implications not only as a scientific milestone but also as a real contribution to humanity [77]. This sequenced genome provided a detailed “blueprint” of the DNA code; however, the machinery that determines when and where individual genes are activated remained to be clarified.

To address this gap, scientists initiated the Human Epigenome Project (HEP) in the 2000s. This initiative aimed to systematically annotate the methylation status of the entire epigenome as a logical extension of the HGP. Central to this approach was an emphasis on epigenetic regulation, which sought to understand how identical DNA sequences could give rise to diverse cellular phenotypes through chemical modifications [78].

The significance of this lies in recognising that genetics alone cannot explain the complexity of biological regulation, and these epigenetic modifications are essential for gene regulation. The Human Epigenome Project, therefore, marked the start of a new era in genomics research. This development plays a vital role in scientists’ work today and will continue to serve as the foundation for future discoveries [79].

The role of artificial intelligence in this field is more than just a technological addition: it represents the next stage of predictive and preventive medicine. Epigenomic information is highly multidimensional, comprising millions of marks, and traditional statistics cannot handle it all. Consequently, with millions of data points per individual, conventional statistical methods often fail to work with this epigenomic data. Therefore, AI helps interpret these vast data collections, aiming to identify patterns and epigenetic markers associated with disease.

This approach enables early identification of disease predispositions before symptom onset. By combining AI-based epigenomic analyses with clinical data, one can not only create personalised treatment plans but also predict how a disease may progress over time. In other words, medicine could finally move away from its traditional reactive approach of waiting for symptoms to develop and then treating them, towards a proactive approach where doctors can intervene before a disease even manifests. Table 2 provides an overview of how AI approaches have been applied to epigenetic data, showing their capacity to predict disease outcomes and identify biomarkers.

In summary, this suggests that individuals at varying levels of risk of developing asthma, COPD, or cancer based on their genetic and epigenetic signature could be identified early, monitored closely, and given targeted advice or treatments to reduce the risk before illness ever manifests. Over time, doctors will have access to the complete genetic and epigenetic profiles of all their patients. This will enable them not only to make accurate diagnoses based on current symptoms but also to determine the precise medication and dosages for each person. With AI-enabled analysis of the data, doctors will be guided on how to care for their patients, ensuring the correct prescriptions are issued, which will save time, reduce suffering, and improve patient outcomes.

Given the above, when AI is introduced into genetic and epigenetic medicine, it will make health a more personalised science. Doctors will not be confined to coming up with guidelines or population-level statistics. Instead, they will be able to view the patient as a special case of unique biology and circumstance. In the future, we hope that the role of medicine will shift towards enhancing survival, refining the quality of life, and providing protection not only for the patient but also for their descendants, thereby addressing the potentially severe repercussions of exposure to detrimental factors.

Most AI studies in this field are based on modest sample sizes, which increases the risk of overfitting and reduces generalizability. Technical variability across laboratories can introduce batch effects, which may distort methylation measurements. Additionally, models are rarely validated across ancestries or external populations, increasing the risk of domain shift. This is particularly important for multigenerational analyses, where epigenetic signatures can be sensitive to population structure, environmental background, and exposure heterogeneity.

## 5. Conclusions

Cigarette smoking has a profound and enduring effect on respiratory health, impacting not only the individual directly exposed but also offspring in subsequent generations through numerous epigenetic mechanisms such as DNA methylation, histone modifications, and non-coding RNA regulation [1,2,3,4,5,6,7,8,9,10,11,12,13,14,15,16,17,18,19,20,21,22,23,24,25,26,27,28,29,30,31,32,33,34,35,36].

The cumulative human evidence and animal models, particularly the work of Rehan et al., suggest that exposure to environmental factors, even before conception or during pregnancy, carries transgenerational risks of lung disease and altered gene expression patterns [33,34]. These molecular changes serve as a memory of environmental trauma, maintaining an increased risk of disease in the offspring of parents who were themselves exposed.

The combination of high-throughput genomic and epigenomic profiling can now be performed alongside AI, providing not only an unprecedented ability to interpret complex data but also new opportunities for identifying predictive biomarkers or modelling disease risk at the individual level [25,26,27,28,29,30,31,32,33,34,35,36,37,38,39,40,41,42,43,44].

Such an amalgam of AI and epigenetics not only may enable earlier detection but could also support more precise prevention of respiratory diseases, including asthma, COPD, and lung cancer, which affect multiple generations. It shields people of all ages from the heavy burden caused by tobacco inheritance [42,43,44,45,46,47,48].

Ultimately, understanding and addressing the epigenetic effects of smoking shifts us from a reactive to a proactive approach to prevention, one that emphasises molecular understanding as a basis, alongside ethical responsibility. The translational potential of AI-driven epigenetic analysis envisages a future where predictive medicine is practised across generations, offering a scientifically informed method to reduce respiratory illnesses and improve public health [62,63].

## Figures and Tables

**Table 1:** Epigenetic changes induced by smoking and their associated disease risks or effects.

Gene/Element	Epigenetic Change Induced by Smoking	Related Disease Risk/Effect
AHRR	DNA hypomethylation at cg05575921;	Biomarker of smoking exposure; associated with lung cancer, COPD, altered lung function
ADAM33	DNA methylation changes in airway epithelial cells	Asthma susceptibility, airway remodelling, and reduced lung function
IL13	DNA methylation changes	Asthma, allergic airway inflammation
SERPINA1	DNA methylation changes	COPD, impaired lung function
PPAR-γ	Histone modification; suppressed expression after smoke exposure	Impaired alveolar development, intergenerational lung pathology
Histone H3	Acetylation induced by tobacco smoke	Activation of pro-inflammatory genes, chronic lung inflammation
miR-21, miR-223	Dysregulated expression due to smoking	Airway inflammation, fibrosis, altered airway responsiveness, transgenerational effects
FosB	Histone acetylation in reward-related brain regions	Altered neuronal plasticity, increased susceptibility to nicotine addiction

**Table 2:** This table illustrates representative applications of artificial intelligence in analysing epigenetic and multi-omic data related to smoking exposure. For each application, the input data type, AI approach, and the resulting predictive outcomes are presented. The table highlights how AI methods—ranging from machine learning classifiers and epigenetic clocks to deep learning models—are employed to predict disease risk, biological ageing, and identify novel epigenetic biomarkers, as well as to detect offspring at risk due to prenatal exposure.

Application	Input Data	AI Approach	Predictive Outcome
Lung disease risk	DNA methylation, histone marks, ncRNA	Machine learning classifiers	Early identification of asthma, COPD, lung cancer risk
Biological ageing	DNA methylation	Epigenetic clocks (GrimAge, GrimAge2)	Estimation of biological age, prediction of age-related disease
Epigenetic biomarker discovery	Multi-omic datasets	Deep learning models	Identification of novel predictive markers for respiratory and neurological diseases
Prenatal exposure effects	DNA methylation in fetal tissues	Predictive modeling	Detection of offspring at risk from maternal smoking exposure

## Data Availability

No new data were created or analyzed in this study.

## Abbreviations

ADAM33	A Disintegrin And Metalloprotease 33
AHRR	Aryl-Hydrocarbon Receptor Repressor
AI	Artificial Intelligence
BAL	Bronchoalveolar Lavage
cg05575921	CpG site 05575921 (in AHRR gene)
COPD	Chronic Obstructive Pulmonary Disease
DNA	Deoxyribonucleic Acid
ECV	Electronic Cigarette Vapour
EPIC-Seq	Enhanced Pooled ImmunoCapture Sequencing
ETS	Environmental Tobacco Smoke
FosB	FBJ Murine Osteosarcoma Viral Oncogene Homolog B
H3	Histone H3
HEP	Human Epigenome Project
HGP	Human Genome Project
IL13	Interleukin 13
lncRNA	Long Non-Coding RNA
MCS	Maternal Cigarette Smoke
miR-21	MicroRNA 21
miR-223	MicroRNA 223
miRNA	MicroRNA
NAcc	Nucleus Accumbens
PAH	Polycyclic Aromatic Hydrocarbons
PPAR-γ	Peroxisome Proliferator-Activated Receptor-γ
RNA	Ribonucleic Acid
SERPINA1	Serpin Peptidase Inhibitor, Clade A, Member 1
